# Lipoprotein(a) as a Predictor of Major Adverse Cardiovascular Events in Patients with Acute Coronary Syndrome

**DOI:** 10.3390/jcm15156086

**Published:** 2026-08-05

**Authors:** Xinhang Li, Huidi Zhang, Jiaxu Bai, Shuai Shi, Chaoyu Sun

**Affiliations:** Department of Cardiology, The Fourth Afffliated Hospital of Harbin Medical University, Harbin 150001, China; 18186878507@163.com (X.L.); 18846057094@163.com (H.Z.); 13636862985@163.com (J.B.)

**Keywords:** Lipoprotein(a), acute coronary syndrome, prognosis, major adverse cardiovascular events, risk prediction, decision curve analysis

## Abstract

**Background**: Despite recent advances in reperfusion strategies, acute coronary syndrome (ACS) patients remain at high risk for recurrent major adverse cardiovascular events (MACE). This study aims to investigate the association between lipoprotein(a) (Lp(a)) and MACE in ACS patients, and to systematically evaluate its predictive performance, incremental prognostic value and clinical utility. **Methods**: This retrospective cohort study included 300 ACS patients with a 1-year follow-up. Serum Lp(a) levels were measured by immunoturbidimetry using fasting venous blood samples collected on the day of admission. Kaplan–Meier and Cox regression analyses were used to assess the association between Lp(a) and MACE. Subgroup and interaction analyses were performed to test the robustness of the findings. Incremental predictive value and net clinical benefit were evaluated using receiver operating characteristic (ROC) curves, the DeLong test, decision curve analysis (DCA) and the integrated discrimination improvement (IDI) index. **Results**: Baseline Lp(a) levels were significantly higher in the event group (*p* = 0.006). A significantly lower cumulative survival probability was found in patients with elevated Lp(a) (log-rank *p* = 0.0014). As a standalone predictor of MACE, Lp(a) yielded an AUC of 0.606 (*p* = 0.006). The AUC of the baseline model was 0.753 (95% CI: 0.694–0.812), which increased to 0.771 (95% CI: 0.712–0.831) after adding Lp(a). DCA showed that the Lp(a)-guided strategy provided higher net benefit than both the “treat-all” and “treat-none” strategies within a threshold probability range of 0–0.35. **Conclusions**: Although Lp(a) offers limited incremental value over traditional risk factors and the Gensini score in the low-to-moderate risk range (<0.35), it provides net clinical benefit as a complementary biomarker in ACS.

## 1. Introduction

Atherosclerotic cardiovascular disease (ASCVD), of which coronary atherosclerotic heart disease (CHD) is a major component, is a highly lethal and disabling condition. A persistent upward trend in the incidence of CHD has been demonstrated [1,2]. CHD results from the interplay between genetic predisposition and environmental factors, among which cold exposure is recognized as a key environmental contributor. Both acute and chronic exposure to cold environments have been associated with increased incidence and mortality of CHD [3,4]. Cities where the daily mean temperature remains below 0 °C for up to three months per year are typically defined as cold-climate cities, hereafter referred to as “cold regions.” Harbin, a representative cold-region city in China, has been reported to have the highest nationwide incidence of cardiovascular disease [5].

Acute coronary syndrome (ACS) represents the acute clinical manifestation of CHD. Its pathogenesis is primarily attributed to thrombus formation triggered by the rupture or erosion of unstable atherosclerotic plaques, leading to acute myocardial ischemia [6]. Effective treatment for ACS patients has been enabled by the widespread adoption of reperfusion therapies—including percutaneous coronary intervention (PCI) and coronary artery bypass grafting (CABG)—along with the establishment of specialized chest pain centers. However, the 2022 Clinical Consensus Statement on Lipid-Lowering Therapy in the Acute Phase of ACS, jointly issued by the European Society of Cardiology and its affiliated societies, confirmed that the first three months following an ACS event constitute the period of highest risk for recurrent cardiovascular events [7]. A substantial proportion of patients continue to experience major adverse cardiovascular events (MACE) during follow-up, including cardiac death, non-fatal MI (Myocardial infarction), recurrent unstable angina, repeat revascularization, and heart failure. Dyslipidemia is a major risk factor among these [8].

Lipoprotein(a) (Lp(a)), a unique plasma lipoprotein first described by Berg in 1963, consists of apolipoprotein(a) covalently linked to a low-density lipoprotein (LDL)-like particle containing apolipoprotein B-100 (ApoB-100) via a disulfide bond [9]. Its unique structural components confer pro-atherosclerotic, pro-thrombotic, pro-inflammatory, and pro-oxidative properties. The 2022 European Atherosclerosis Society consensus statement explicitly stated a causal relationship between Lp(a) and ASCVD [10]. The 2024 ESC Guidelines clearly state that elevated Lp(a) is a marker of cardiovascular risk, particularly for early-onset atherosclerotic disease [11]. The EAS consensus statement defines Lp(a) ≥ 50 mg/dL (mass concentration) or ≥125 nmol/L (molar concentration) as a cardiovascular risk-enhancing factor. It further notes that Lp(a) levels are significantly influenced by race, with racial differences primarily attributable to variations in the LPA gene locus rather than external factors; consequently, Lp(a) is regarded as a stable and reproducible risk factor. Although traditional biomarkers remain essential for assessing cardiometabolic risk [12], they are distinct from the genetically determined cardiovascular risk captured by Lp(a) [10].

Existing observational studies have established an association between Lp(a) and prognosis in ACS patients [10,11]. However, previous investigations have mostly been limited to univariate or multivariate Cox regression analyses, and the incremental predictive value of Lp(a) beyond traditional risk models has rarely been systematically evaluated. Moreover, the Gensini score—a well-validated quantitative indicator of coronary artery disease severity—is a strong predictor of long-term prognosis in ACS patients, but its role in incremental risk assessment when combined with traditional models has received limited attention [13]. In parallel, whether Lp(a) provides net clinical benefit for guiding therapeutic decisions remains to be clarified, particularly given the paucity of evidence specific to the Chinese patients with ACS.

Given this background, the present study adopted a retrospective cohort design to investigate the association between baseline Lp(a) levels and MACE in Chinese patients with ACS, aiming to clarify its independent prognostic value. We assessed the robustness of the findings through subgroup analyses and interaction tests, and systematically evaluated discriminative ability, incremental predictive value, and clinical net benefit. Collectively, this study provides clinical practice-based evidence for integrating Lp(a) into risk assessment for ACS patients.

## 2. Materials and Methods

### 2.1. Study Design and Population

A retrospective, observational study was conducted at the Fourth Affiliated Hospital of Harbin Medical University. A total of 300 consecutive patients admitted with ACS were enrolled between January 2024 and December 2024. The inclusion criteria were as follows: (1) chest pain with Coronary angiography (CAG) performed; (2) ACS diagnosed according to the Guidelines for the Treatment of Acute Coronary Syndrome; and (3) age > 18 years. The exclusion criteria were as follows: (1) primary or secondary cardiomyopathy, valvular heart disease, or heart failure; (2) prior ACS with PCI or CABG; (3) severe hepatic or renal insufficiency, thyroid dysfunction, malignancy, or immune-related diseases; (4) history of stroke; and (5) incomplete baseline data. A total of 387 patients with ACS were initially screened. After applying the exclusion criteria, 87 patients were excluded (most commonly due to multiple comorbidities [*n* = 38], prior PCI/CABG [*n* = 20], and incomplete baseline data [*n* = 29]), and 300 patients were finally enrolled in the analysis. To reduce potential confounding effects, this study excluded patients with multiple comorbidities or those with known factors that affect Lp(a) levels. This approach aims to obtain a more homogeneous study population, thereby enabling a clearer assessment of the association between Lp(a) and the primary outcome indicators. This is consistent with the recommended practices of previous similar studies [14].

All patients received standard ACS medical therapy according to current guidelines, including dual antiplatelet therapy, statins, beta-blockers, and ACEI/ARB as clinically indicated [15]. For patients with elevated Lp(a), intensified secondary prevention—including stricter LDL-C targets and comprehensive risk factor management—may be considered. In addition, proprotein convertase subtilisin/kexin type 9 inhibitors (PCSK9i) (e.g., evolocumab, alirocumab) have been shown to reduce Lp(a) levels by approximately 20–30% [16,17].

### 2.2. Data Collection

Baseline patient data were collected via the electronic medical record system, including demographics [sex, age, body mass index (BMI)]; general clinical data [smoking history (current or cumulative smoking ≥6 months), and past medical history (hypertension, diabetes)]; laboratory data [Lp(a), apolipoprotein A1 (apo A1), apolipoprotein B (apo B), total cholesterol (TC), triglycerides (TG), high-density lipoprotein cholesterol (HDL-C), low-density lipoprotein cholesterol (LDL-C), serum creatinine, blood urea nitrogen, alanine aminotransferase (ALT), aspartate aminotransferase (AST), and cardiac enzymes]; and imaging data [left ventricular ejection fraction (LVEF) assessed by echocardiography]. CAG was performed and interpreted by an interventional cardiologist, with coronary artery lesions scored using the Gensini scoring system. This score is calculated based on the degree of coronary artery stenosis (1 point for ≤25%, 2 points for 26–50%, 4 points for 51–75%, 8 points for 76–90%, 16 points for 91–99%, 32 points for 100%), multiplied by the corresponding weight coefficient for each vascular segment (left main artery × 5, proximal left anterior descending artery × 2.5, middle segment × 1.5, distal segment × 1.0, proximal circumflex artery × 2.5, distal segment × 1.0, right coronary artery × 1.0, corresponding coefficients for diagonal branch and posterior branch). The sum of the scores for each segment is the total Gensini score [18]. Lp(a) levels were determined by immunoturbidimetric assay from venous blood samples collected after an overnight fast. Based on Lp(a) levels, patients were classified into a normal group (<30 mg/dL) and an elevated group (≥30 mg/dL). Stratified analyses were also performed according to Lp(a) quartiles (Q1, lowest; Q4, highest).

### 2.3. Endpoints

The primary endpoint was MACE, defined as a composite of cardiac death, non-fatal MI, recurrent unstable angina, repeat revascularization (PCI), and heart failure occurring within one year. Regular outpatient follow-up was scheduled for all patients from the date of discharge and was conducted at 1, 3, 6, and 12 months post-discharge. Endpoint event dates were recorded immediately at occurrence. If the follow-up period of 365 days has not resulted in the occurrence of the endpoint event, then 365 days will be used as the cut-off time for survival analysis.

### 2.4. Statistical Analysis

#### 2.4.1. Normality Tests and Data Presentation

Statistical analyses were performed using SPSS 27.0 (IBM SPSS Statistics 27.0.1). Normality was assessed by the Shapiro–Wilk test. Normally distributed variables were expressed as χ2 ± SD and compared using the independent-samples *t*-test; non-normally distributed variables were expressed as median (P25, P75) and compared using the Mann–Whitney U test. Categorical variables were presented as frequencies (%) and compared using the chi-square test (χ2).

#### 2.4.2. Survival Analysis

Survival analyses were performed using R software (version 4.4.3). Kaplan–Meier survival curves were generated for patients stratified according to Lp(a) dichotomization (normal vs. elevated) and Lp(a) quartiles (Q1 to Q4). Differences in survival distributions between groups were compared using the log-rank test.

#### 2.4.3. Univariate and Multivariate Cox Regression Analysis

Univariate Cox regression analysis was performed using SPSS 27.0 to identify variables associated with the endpoint. Subsequently, multivariate Cox regression models were constructed with adjustment for age, sex, smoking history, hypertension, diabetes, LVEF, Gensini score, and relevant laboratory parameters, while variables exhibiting multicollinearity were excluded. The independent effect of Lp(a) on the endpoint was assessed using multivariate Cox regression. Collinearity was evaluated using the variance inflation factor (VIF) and tolerance. A VIF > 5 or tolerance < 0.2 was considered to indicate moderate collinearity, whereas a VIF > 10 or tolerance < 0.1 indicated severe collinearity; variables with moderate or higher levels of collinearity were excluded from the final models.

#### 2.4.4. Assessment of Predictive Value

The development and reporting of the prediction model followed the Transparent Reporting of a multivariable prediction model for individual prognosis or diagnosis (TRIPOD) statement [19]. ROC curves were generated using R software based on the predicted probabilities derived from the base model and the extended model. The area under the curve (AUC) and its 95% confidence interval (CI) were calculated for each model. The DeLong test was applied to compare the AUCs of the two models, and the AUC for the univariate Lp(a) model was analyzed separately. A univariate logistic regression model, with Lp(a) as the sole predictor, was used to estimate the predicted probability of endpoint events for each patient. DCA was performed in R to evaluate the net clinical benefit of an Lp(a)-guided intervention across a threshold probability range of 0 to 1, compared with the “treat-all” and “treat-none” strategies. The IDI index was calculated to quantify the incremental predictive value of adding Lp(a) to the base model.

#### 2.4.5. Subgroup Analysis and Interaction Tests

Subgroup analyses were performed using SPSS 27.0, with stratification by sex, age, and ACS classification. Within each subgroup, the multivariate Cox regression model was rerun with adjustment for the same covariates as those included in the main model to estimate the hazard ratio (HR) and 95% CI for Lp(a). Interaction tests were subsequently conducted, and a two-sided *p* value < 0.05 was considered to indicate a statistically significant interaction.

## 3. Results

### 3.1. Baseline Characteristics

**Figure 1:**
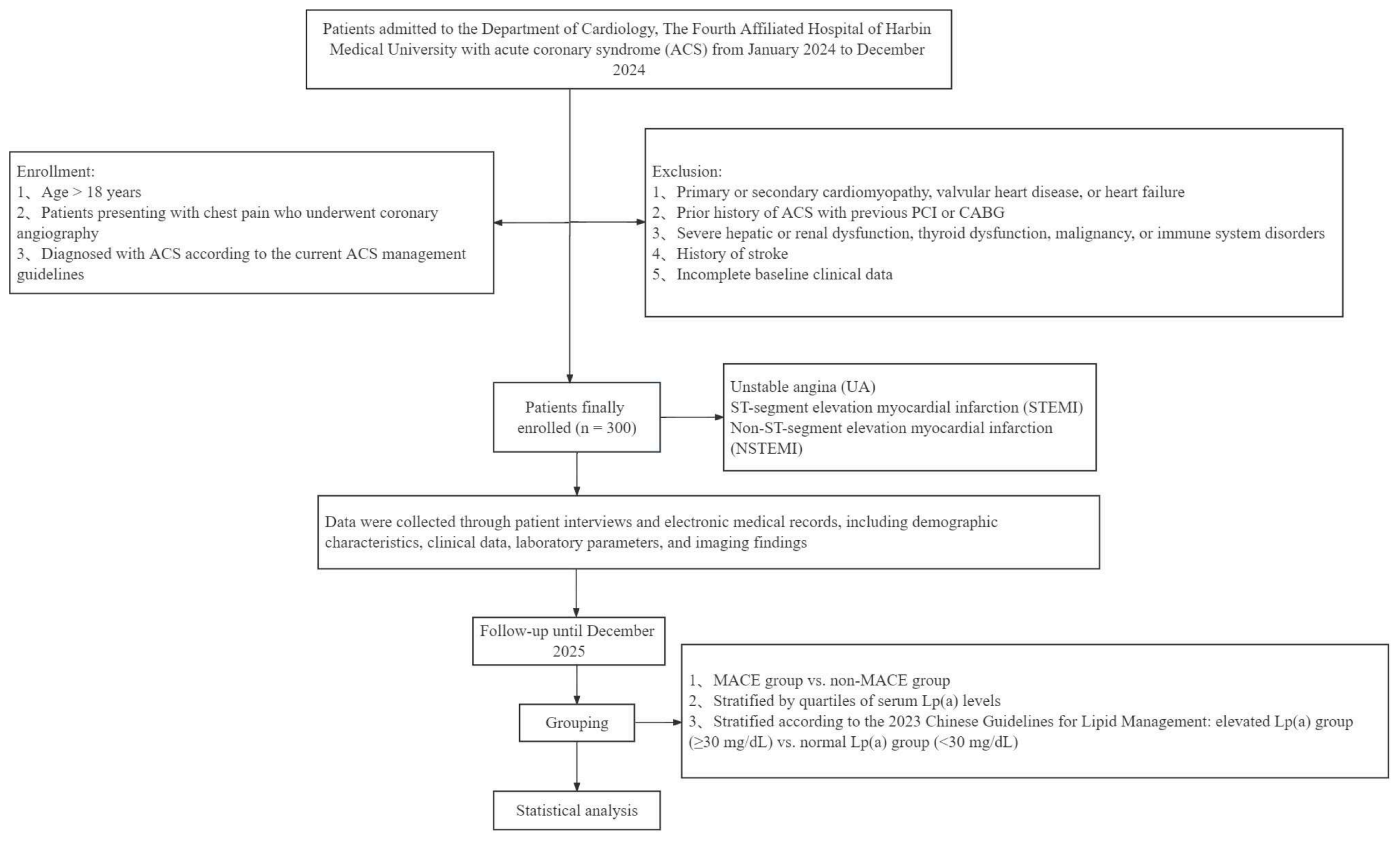
Flowchart of patient enrollment and study design. Abbreviations: ACS, acute coronary syndrome; PCI, percutaneous coronary intervention; CABG, coronary artery bypass grafting; MACE, major adverse cardiovascular events.

The patient enrollment and screening process is shown in Figure 1. Of 387 patients screened, 300 were included in the final analysis. During follow-up, 76 patients (25.3%) experienced endpoint events. Significant differences between the event and non-event groups were observed for sex, smoking history, Lp(a) levels, Gensini score, and ACS subtype (all *p* < 0.05) (Table 1). Baseline demographic, clinical, and laboratory characteristics of the study population are summarized in Table 1.

### 3.2. Univariate and Multivariate Cox Regression Analysis

In univariate analysis, male sex, smoking history, white blood cell count, creatine kinase, Lp(a), LVEF, and Gensini score were significantly associated with the endpoint (all *p* < 0.05). In the multivariate analysis, after adjusting for potential confounders and excluding variables with multicollinearity (VIF > 5), both Lp(a) (per 1 mg/dL increase: HR = 1.021, 95% CI: 1.011–1.031, *p* < 0.001) and Gensini score (per 1-point increase: HR = 1.016, 95% CI: 1.010–1.022, *p* < 0.001) remained independent risk factors for endpoint events (Table 2).

### 3.3. Subgroup and Interaction Analyses

**Figure 2:**
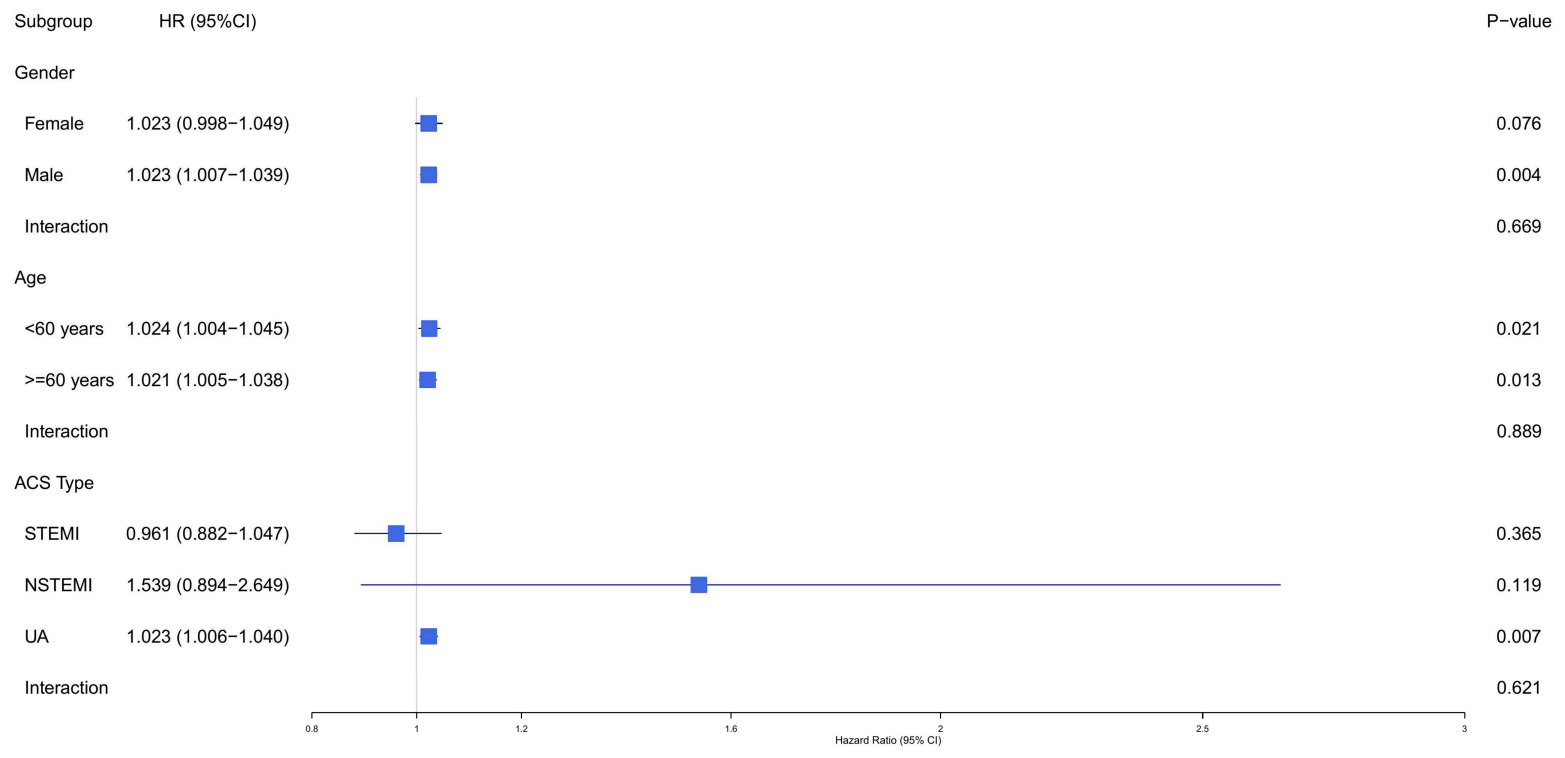
Subgroup Analysis: Lp(a) and MACE in ACS Patients. Abbreviations: ACS, acute coronary syndrome; STEMI, ST-segment elevation myocardial infarction; NSTEMI, non-ST-segment elevation myocardial infarction; UA, unstable angina.

In Subgroup analyses revealed that the association between Lp(a) and endpoint events was statistically significant in male patients (59 cases, HR = 1.023, 95% CI: 1.007–1.039, *p* = 0.004), in patients aged <60 years (31 cases, HR = 1.024, 95% CI: 1.004–1.045, *p* = 0.021), in patients aged ≥60 years (45 cases, HR = 1.021, 95% CI: 1.005–1.038, *p* = 0.013), and in patients with UA (45 cases, HR = 1.023, 95% CI: 1.006–1.040, *p* = 0.007). None of the interaction tests reached statistical significance (all *p* > 0.05) (Table 3; Figure 2). However, it did not reach statistical significance in the female subgroup (17 cases), the STEMI subgroup (9 cases), and the NSTEMI subgroup (22 cases).

### 3.4. Survival Curves

**Figure 3:**
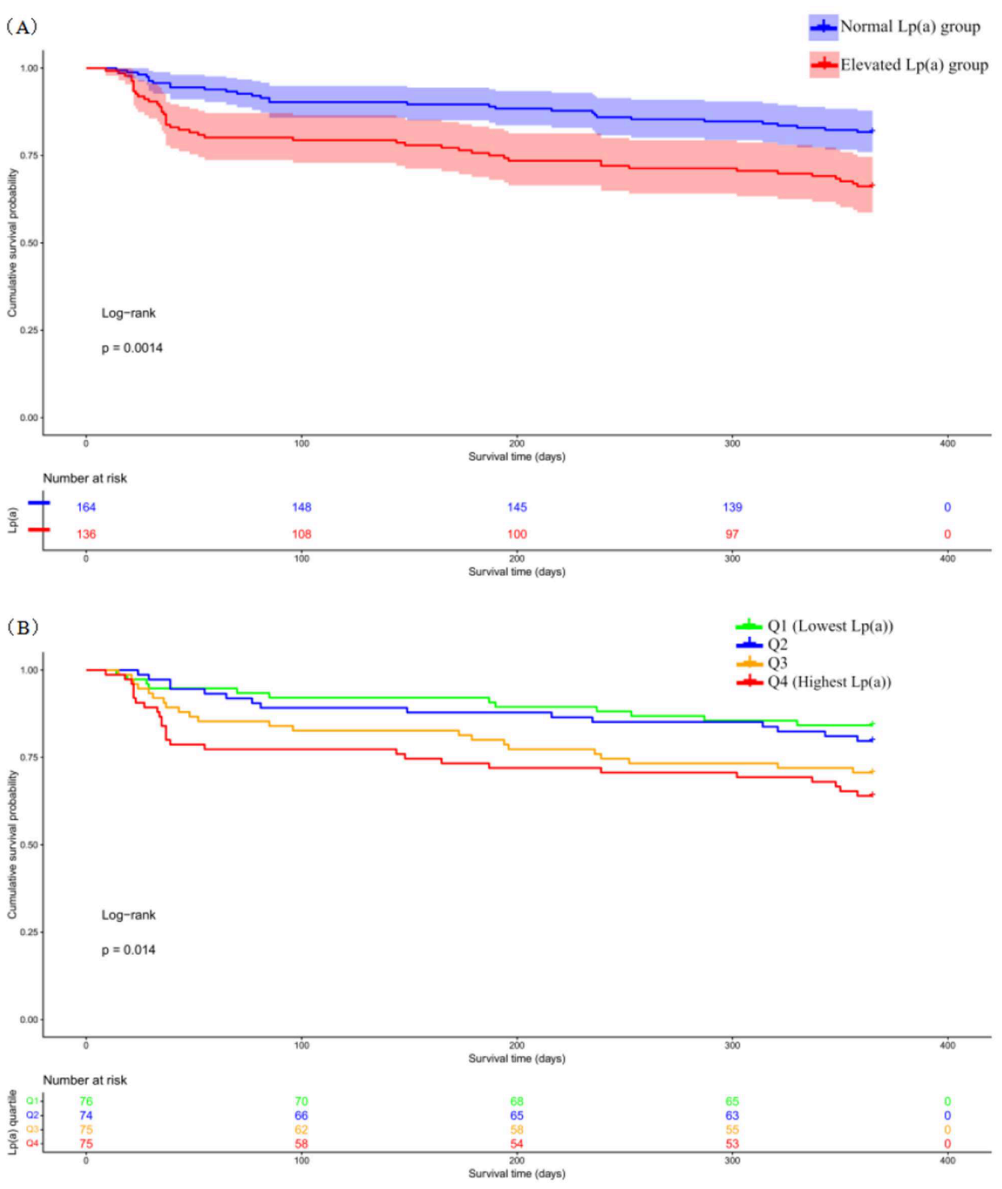
(**A**) Kaplan–Meier survival curves stratified by dichotomous lipoprotein(a) (Lp(a)) levels. (**B**) Kaplan–Meier survival curves stratified by Lp(a) quartiles (Q1–Q4).

The elevated Lp(a) group demonstrated a significantly lower 1-year cumulative survival rate (log-rank *p* = 0.0014) (Figure 3A). Quartile-based analysis further demonstrated a progressive decline in survival rates with increasing Lp(a) levels (log-rank *p* = 0.014) (Figure 3B).

### 3.5. ROC Curves

**Figure 4:**
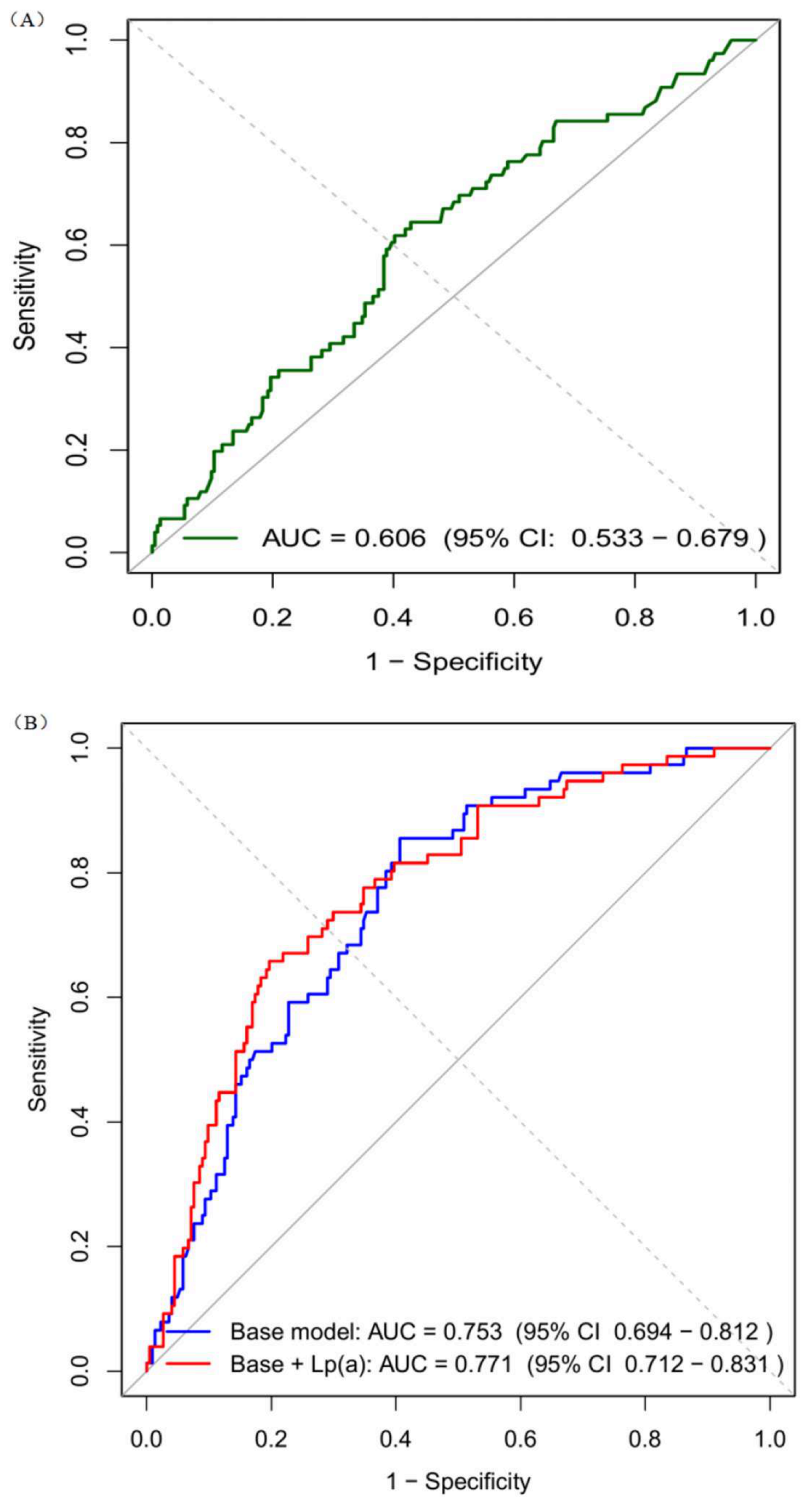
(**A**) Receiver operating characteristic (ROC) curve of univariate Lp(a). (**B**) Receiver operating characteristic (ROC) curves of the baseline model versus the baseline model plus Lp(a). The dashed diagonal line indicates the reference line (AUC = 0.5). Abbreviations: AUC, area under the curve; CI, confidence interval.

The AUC for Lp(a) alone in predicting endpoint events was 0.606 (95% CI: 0.533–0.679, *p* = 0.006) (Figure 4A). The AUC of the baseline model, which incorporated traditional risk factors and the Gensini score, was 0.753 (95% CI: 0.694–0.812) and increased to 0.771 (95% CI: 0.712–0.831) after the addition of Lp(a) (DeLong test *p* = 0.237) (Figure 4B). The optimal cut-off value was determined to be 29.65 mg/dL using the method of maximizing the Youden index, with a corresponding sensitivity of 61.8% and specificity of 59.8%.

### 3.6. DCA and IDI

**Figure 5:**
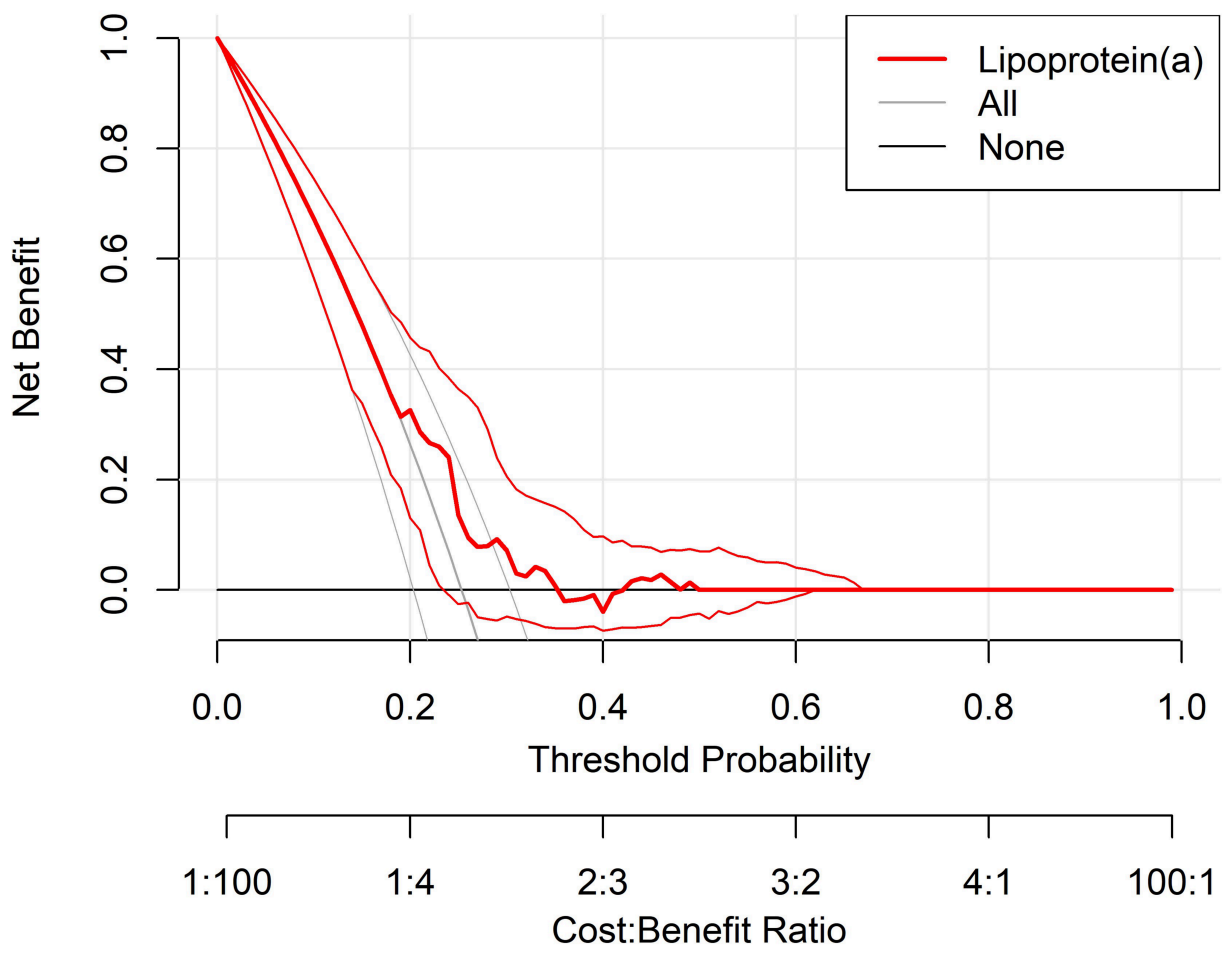
Decision curve analysis of the univariate lipoprotein(a) model.

DCA demonstrated a superior net benefit for the Lp(a)-based model over the “treat-all” and “treat-none” strategies within a threshold probability range of 0 to 0.35 (Figure 5). A baseline model (Model 2) incorporating traditional cardiovascular risk factors was constructed, and an extended model (Model 3) was developed by adding Lp(a) to Model 2. Compared with the baseline model, the extended model yielded an IDI of 0.0172 (95% CI: −0.0068 to 0.0400, *p* = 0.180) (Table 4).

## 4. Discussion

### 4.1. Main Findings

In this study, Lp(a) was demonstrated to be an independent risk factor for composite endpoint events within one year in patients with ACS. As presented in Table 1, the endpoint event group had a higher proportion of male patients (77.6% vs. 59.8%, *p* = 0.005) and current smokers (34.2% vs. 21.4%, *p* = 0.026). Lp(a) levels were significantly elevated in the event group (median: 33.3 mg/dL vs. 18.25 mg/dL, *p* = 0.006), and Gensini scores were also markedly higher (median: 63.5 vs. 23.5, *p* < 0.001). Regarding ACS subtype distribution, the event group had a higher proportion of NSTEMI (29.0%) and unstable angina (UA; 59.2%), whereas no endpoint events occurred in patients with Cardiac Syndrome X (0% vs. 13.8%; *p* < 0.001). In the multivariate analysis, after adjustment for potential confounders and exclusion of variables with multicollinearity, the final model identified Lp(a) and the Gensini score as independent risk factors for endpoint events.

These findings are consistent with numerous clinical studies confirming that Lp(a) provides prognostic information beyond traditional risk factors and reflects residual cardiovascular risk. For instance, Liu et al. reported a 2.1-fold increased risk of recurrent events in patients with coronary heart disease and Lp(a) levels ≥30 mg/dL (HR = 2.1) [20]. In the present study, Lp(a) was analyzed as a continuous variable, and each 1 mg/dL increase corresponded to an HR of 1.021, a trend consistent with Liu et al.’s findings. In a cohort of 12,064 patients undergoing PCI, Yoon et al. confirmed that Lp(a) levels >30 mg/dL were associated with a 25% increased risk of recurrent ischemic events (HR = 1.25) [21]. Our study observed a similar direction of effect in ACS patients, with significant differences evident even with a relatively short follow-up period (1 year), suggesting that the prognostic impact of Lp(a) may manifest early. A meta-analysis by Wang et al., which pooled data from 18,168 ACS patients, reported a 26% increased risk of MACE associated with elevated Lp(a) levels (HR = 1.26) [22]. Our findings are consistent with this meta-analysis, further supporting the value of Lp(a) as a risk stratification tool in ACS patients. Furthermore, a systematic review by Mojahedi et al., encompassing 10 studies and 20,896 patients, concluded that Lp(a) serves as a potent predictive biomarker for recurrent ischemic events in ACS patients following PCI, with HRs ranging from 1.14 to 4.29 [23].

As presented in Table 3 and Figure 2, the prognostic effect of Lp(a) was statistically significant in male patients, in those aged <60 years, in those aged ≥60 years, and in patients with UA. In contrast, the HR estimates did not reach statistical significance in female patients, those with STEMI, or those with NSTEMI (all *p* > 0.05). However, the number of endpoint events in these subgroups was relatively small (17 events in females, 9 in STEMI, and 22 in NSTEMI), resulting in insufficient statistical power (below 80%). Consequently, these negative findings may reflect inadequate sample size rather than a true absence of an Lp(a) effect. Larger, well-powered studies—such as multi-center cohorts or meta-analyses—are warranted to reliably assess the potential prognostic impact of Lp(a) in these subgroups. Notably, none of the interaction tests reached statistical significance (sex: *p* = 0.669; age: *p* = 0.889; ACS subtype: *p* = 0.621), indicating that the effect of Lp(a) on endpoint events did not differ significantly across strata of sex, age, or ACS subtype. These findings support the robustness and generalizability of the observed associations. Our results are consistent with previous reports. Qin et al. demonstrated significantly lower all-cause and cardiovascular mortality in the low Lp(a) group compared with the high Lp(a) group [24]. Similarly, a Korean study of patients with AMI by Park et al. reported a significantly higher 3-year incidence of MACE in the Lp(a) ≥ 50 mg/dL group than in the Lp(a) < 30 mg/dL group (*p* < 0.001) [25].

As shown in Figure 3A, the elevated Lp(a) group had a significantly lower 1-year cumulative survival rate than the normal Lp(a) group. The survival curves of the two groups diverged early during follow-up, and the curve for the elevated group remained consistently below that of the normal group throughout the study period. The median survival time in the normal Lp(a) group was 325.220 days (95% CI: 310.280–340.159), significantly longer than the 281.375 days (95% CI: 258.643–304.107) observed in the elevated group. The log-rank test confirmed a statistically significant difference in survival distributions between the two groups (*p* = 0.0014). Quartile-based analysis of Lp(a) levels is presented in Figure 3B. The survival curves demonstrated a progressive decline in cumulative survival rates with increasing Lp(a) levels. Survival was consistently highest in the Q1 group and lowest in the Q4 group. For instance, at the end of follow-up (365 days), survival rates of 0.90, 0.91, 0.88, and 0.86 were observed for the Q1, Q2, Q3, and Q4 groups, respectively. The log-rank test indicated that the overall differences in survival curves among the four groups were statistically significant (χ2 = 10.655, *p* = 0.014). These findings further support a dose–response relationship between Lp(a) levels and poor prognosis. The number-at-risk table confirmed complete follow-up data, supporting the reliability of these results. Consistent with our findings, Li et al. reported a significantly higher cumulative incidence of MACE in the high Lp(a) group (*p* < 0.001) in a cohort of 1504 patients with ACS and three-vessel disease, with an AUC of 0.623 for Lp(a) in predicting MACE. Additionally, ROC analysis revealed an approximately U-shaped nonlinear association between log-transformed Lp(a) (log10Lp(a)) and MACE risk (*p* for nonlinearity < 0.001) [26]. Our study extends these observations to the general ACS population without three-vessel disease and obtained similar findings with respect to survival curve separation. Jung et al. compared the predictive performance of the ApoB/A1 ratio, TC/HDL-C ratio, and Lp(a) for post-PCI MACE. They reported that Kaplan–Meier curves stratified by Lp(a) tertiles could significantly discriminate MACE risk, indicating that Lp(a) offers certain prognostic advantages over conventional lipid markers [27]. In the present study, the Kaplan–Meier curve using a cutoff of 30 mg/dL also demonstrated significant discrimination (*p* = 0.0014), supporting the role of Lp(a) as a risk marker independent of the traditional lipid profile.

From a pathophysiological perspective, Lp(a) may increase plaque instability and the risk of ischemic events through multiple mechanisms, including impairment of endothelial function, promotion of intraplaque lipid accumulation, enhancement of local inflammatory responses, and inhibition of fibrinolytic activity [28]. A comprehensive review by Hussain et al. elucidated the role of Lp(a) in cardiovascular disease within the Chinese population, emphasizing ethnic differences in Lp(a) levels and its associations with atherosclerosis, thrombosis, and inflammation [29]. Similarly, the New Zealand Multi-Ethnic Study of Acute Coronary Syndromes (MENZACS) demonstrated marked ethnic variations in Lp(a) levels, with the predictive value of Lp(a) being present across all ethnic groups (HR for the highest vs. lowest quartile: 1.46) [30]. The present study further confirms the prognostic value of Lp(a) in the Han Chinese population, suggesting that this biomarker possesses cross-ethnic generalizability. Importantly, the high consistency between our clinical observations and the aforementioned mechanistic pathways provides a robust biological rationale for the prognostic value of Lp(a).

A ROC curve was constructed with the endpoint event as the response variable and Lp(a) as the predictor variable. As shown in Figure 4A, an AUC of 0.606 was obtained, indicating that Lp(a) possesses modest predictive value for the endpoint event. Based on previous literature on ACS prognosis, the following recognized predictors were incorporated into the baseline model: age, sex, smoking history, hypertension, diabetes, and Gensini score. These variables are well-established risk factors for adverse cardiovascular outcomes and are routinely adjusted for in multivariate analyses. The baseline model plus Lp(a) constituted the extended model. As presented in Figure 4B, the baseline model yielded an AUC of 0.753 (95% CI: 0.694–0.812), whereas the AUC of the extended model increased to 0.771 (95% CI: 0.712–0.831). Both AUCs were significantly greater than 0.5 (*p* < 0.001), representing an absolute increase of 0.018. The DeLong test for the difference between the two AUCs yielded a Z value of −1.1819 (*p* = 0.2373; 95% CI: −0.0493 to 0.0122), indicating that no statistically significant improvement in discriminative ability was achieved after the addition of Lp(a). Although Lp(a) numerically increased the AUC from 0.753 to 0.771, this increase may merely reflect random fluctuation. Possible explanations include the limited sample size (only 76 events) or the fact that the Gensini score—a well-established, strong predictor—was already incorporated into the baseline model, thereby limiting the ability to detect an incremental effect. Notably, Lp(a) alone yielded an AUC of 0.606 (95% CI: 0.533–0.679, *p* = 0.006), indicating that Lp(a) possesses modest predictive value on its own. The lack of a significant increase in AUC after adding Lp(a) to the baseline model may be attributed to the fact that the prognostic effect of Lp(a) is partially mediated through the promotion of more severe coronary lesions, information already captured by the Gensini score [31]. Consequently, we conclude that the incremental discriminatory power of Lp(a) is limited.

A key indicator for assessing the incremental value of a biomarker is the IDI. As shown in Figure 5, within a threshold probability range of 0 to 0.35, the Lp(a) predictive model showed a significantly higher net benefit than the “treat-all” and “treat-none” strategies, indicating that risk stratification based on Lp(a) levels to guide intervention yields favorable net clinical benefit within this range. When the threshold probability exceeded 0.35, the net benefit of the model gradually diminished, and its clinical utility became limited. Consequently, Lp(a) may serve as an adjunctive biomarker for risk stratification in ACS patients, assisting clinicians in making refined decisions following routine risk assessment, particularly when the estimated risk of endpoint events falls within the 0–35% range. For newly admitted ACS patients, the predicted probability of endpoint events can be calculated using the univariate logistic regression model with Lp(a). If the predicted probability is <0.35, Lp(a) levels should be considered: elevated Lp(a) (>30 mg/dL) would warrant more intensive secondary prevention measures, including aggressive lipid-lowering therapy, optimized antiplatelet therapy, and closer follow-up; normal Lp(a) levels support routine guideline-directed management. Conversely, if the predicted probability is ≥0.35, the net benefit of the Lp(a) model approaches zero, and clinical decisions should be guided primarily by traditional risk factors and the Gensini score, rather than over-relying on Lp(a) levels.

To more comprehensively capture patients’ baseline risk, we constructed a baseline model (Model 2) incorporating traditional cardiovascular risk factors. We subsequently developed an extended model (Model 3) by adding Lp(a) to Model 2. As presented in Table 4, the extended model yielded an IDI of 0.0172 (95% CI: −0.0068 to 0.0400, *p* = 0.180) compared with the baseline model, indicating a numerically improved predictive ability that did not reach statistical significance. Bhatia et al. reported a modest improvement in 10-year ASCVD risk prediction in low-risk individuals (<7.5%) when Lp(a) was added to the AHA PREVENT risk prediction equation in the Multi-Ethnic Study of Atherosclerosis (MESA, 2000–2018; *n* = 6670) and the UK Biobank (UKB, 2006–2022; *n* = 308,113) [32]. These findings are consistent with the present study regarding the role of Lp(a) in reclassifying low-risk individuals. In the BioHEART-CT study, adding Lp(a) to traditional risk factors resulted in a net reclassification improvement (NRI) of 16% (*p* = 0.001) and an IDI of 0.0046 (*p* = 0.03), suggesting that approximately 5% of patients could be correctly reclassified by Lp(a) [33]. In contrast, statistical significance was not achieved in the present study; potential reasons for this discrepancy have been discussed above. Larger cohort studies are warranted to further validate the incremental reclassification value of Lp(a).

### 4.2. Clinical Implications

Our findings indicate that elevated Lp(a) is an independent risk factor for long-term MACE in patients with ACS, with consistent prognostic value demonstrated across sexes, age groups, and ACS subtypes. This suggests that Lp(a) may serve as a universal adjunctive biomarker applicable to a broad ACS population, rather than being limited to specific subgroups. Although Lp(a) offers limited incremental discriminative ability when added to traditional risk models, DCA confirms that it provides meaningful clinical net benefit within the low-to-moderate risk threshold range. Accordingly, Lp(a) testing may further refine risk stratification based on existing assessment systems and complement the limitations of traditional risk factors and CAG evaluation. In clinical practice, Lp(a) represents a simple, stable, and reproducible biomarker for identifying patients with residual cardiovascular risk. Particularly among those with ACS classified as low-to-moderate risk at baseline, elevated Lp(a) levels indicate potential plaque instability and increased thrombotic risk. This facilitates more accurate identification of individuals who may benefit from enhanced follow-up, stricter risk factor control, and optimized lipid-lowering strategies. Consequently, Lp(a) enables more personalized, risk-oriented management and offers practical, meaningful clinical value in improving long-term prognosis and rationally allocating healthcare resources. Currently, no conventional drugs are specifically designed to lower Lp(a) [34]; however, PCSK9i such as evolocumab and alirocumab, can reduce Lp(a) levels by approximately 20–30% [16,17]. In light of these findings, intensified secondary prevention—including stricter LDL-C targets, optimized antiplatelet therapy, and rigorous risk factor control—is warranted for patients with elevated Lp(a).

### 4.3. Strengths and Limitations

#### 4.3.1. Strengths

This single-center retrospective cohort study strictly adhered to predefined inclusion and exclusion criteria. It focused on the prognostic association between Lp(a) and long-term MACE with a clearly defined research question and strong clinical relevance, thereby providing localized evidence for risk assessment in the Chinese patients with ACS. The multivariate analysis not only adjusted for traditional cardiovascular risk factors but also incorporated the Gensini score, a well-established indicator of coronary artery disease severity, effectively controlling for potential confounding bias and strengthening the credibility of the independent prognostic value of Lp(a). This study comprehensively employed Kaplan–Meier survival analysis, Cox regression, subgroup analyses, interaction tests, ROC curves, DCA, and the IDI index to systematically evaluate Lp(a) from multiple dimensions, including independent prognostic value, effect stability, predictive performance, and clinical net benefit. The analytical approach was thorough and bridged statistical associations with clinical decision-making, ensuring that the conclusions were closely aligned with clinical practice. Subgroup and interaction analyses confirmed that the prognostic effect of Lp(a) was generally consistent across sexes, age groups, and ACS subtypes, suggesting that Lp(a) possesses value for risk stratification across diverse ACS populations and that the findings are broadly generalizable. Moreover, the study truthfully reported the limited incremental discriminative ability of Lp(a) while emphasizing its clinical net benefit only in low-to-moderate-risk populations, which aligns with the objective principles of biomarker research and ensures high credibility.

#### 4.3.2. Limitations

This study is a single-center, retrospective observational study that has not been externally validated in an independent cohort, and patients with multiple underlying diseases were excluded. Although this exclusion criterion helps to control confounding factors, it may also introduce selection bias, resulting in systematic differences between the study population and the actual clinical population in the real world. This, to some extent, limits the external validity and general applicability of the research conclusions, which may introduce selection and information biases and consequently limit the generalizability of the findings. Secondly, due to the limitations of the retrospective data collection method, some patients had missing Lp(a) or lipid test results because of incomplete clinical records. The mechanism of this missing data may be related to the patient’s own characteristics (such as the severity of the disease, the department of consultation or the testing habits of the clinical doctor), and it is not completely random missing. This constitutes a potential information bias.

A total of 300 patients were ultimately included, with 76 cases of MACE observed during follow-up. The relatively modest sample size resulted in a limited number of events in certain subgroups (17 events in women, 9 in STEMI, and 22 in NSTEMI), which may have affected the stability of some subgroup analyses. This is likely the primary reason for the null findings in these subgroups, rather than a true lack of prognostic effect of Lp(a). Lp(a) levels were measured only at admission, and dynamic changes during follow-up were not assessed; consequently, the impact of long-term Lp(a) fluctuations on clinical outcomes remains unknown. As an observational prognostic study, this investigation can only establish an independent association between Lp(a) and MACE, and causality cannot be directly inferred. Further exploration through basic research is warranted to elucidate the underlying mechanisms. The predictive model developed in this study has not yet been translated into a convenient clinical tool, limiting its direct point-of-care applicability. Furthermore, the study population was exclusively recruited from Harbin, a cold region in China, and given the well-documented ethnic differences in Lp(a) levels, the generalizability of these findings to non-Asian populations remains unclear.

### 4.4. Future Research Directions

Multicenter prospective cohort studies encompassing more diverse populations (across different ethnicities and climatic regions) are warranted to validate the generalizability of the present findings. Additionally, during the design process, efforts should be made to optimize the data collection procedure as much as possible to minimize the impact of missing data on the robustness of the results. Dynamic monitoring of Lp(a) levels should be undertaken to investigate post-ACS trajectories of Lp(a) changes and their relationship with clinical outcomes. Subgroup-specific studies with larger sample sizes are needed for populations in which statistical power was insufficient in the current study, so that differences in the prognostic value of Lp(a) across various patient groups can be further clarified and more targeted evidence for risk stratification in distinct populations can be provided. With the ongoing clinical development of Lp(a)-lowering agents such as pelacarsen and olpasiran [35,36], future research should focus on randomized controlled trials of Lp(a)-targeted therapies to determine whether lowering Lp(a) translates into a reduction in cardiovascular events and which patient subgroups are most likely to benefit. In addition, prospective interventional studies are needed to provide high-quality evidence for the optimization of clinical treatment strategies. Risk prediction models could be further refined by integrating Lp(a) with existing risk assessment tools, such as the AHA PREVENT model and the GRACE score [37], so that risk stratification strategies better suited to the Chinese patients with ACS can be developed and more precise individualized management enabled.

## 5. Conclusions

Elevated baseline Lp(a) was identified as an independent risk factor for MACE in patients with ACS, but its incremental predictive value beyond conventional risk factors and the Gensini score is limited in the low-to-moderate risk range (<0.35). Nevertheless, Lp(a) provides net clinical benefit as a complementary biomarker, particularly when clinical uncertainty persists after routine assessment. These findings support incorporating Lp(a) into risk stratification to identify patients with residual risk who may benefit from intensified follow-up and risk factor management. Further validation in larger, multicenter prospective cohorts is warranted.

## Figures and Tables

**Table 1:** Baseline characteristics of ACS patients according to endpoint event.

	Event Group (*n* = 76)	Non-Event Group (*n* = 224)	*p* Value
Age, years	61 (54.25, 69.5)	60 (54, 68)	0.521
Sex, *n* (%)			0.005
Male	59 (77.6)	134 (59.8)	
Female	17 (22.4)	90 (40.2)	
Medical history			
BMI, kg/m^2^	25.4 (23.1, 27.0)	25.05 (22.9, 27.3)	0.532
Smoking, *n* (%)	26 (34.2)	48 (21.4)	0.026
Hypertension	37 (48.7)	117 (52.2)	0.593
Diabetes	19 (25.0)	40 (17.9)	0.176
SBP, mmHg	132.5 (120, 146.8)	136 (124, 148)	0.325
DBP, mmHg	81 (74, 87)	82 (75, 89)	0.654
WBC, ×10^9^/L	6.75 (5.29, 8.56)	6.28 (5.31, 7.63)	0.124
Creatinine, μmol/L	73.6 (61.4, 83.6)	67.9 (57.9, 78.1)	0.056
Uric acid, μmol/L	329.8 (283.1, 420.5)	320.9 (260.6, 384.2)	0.079
AST, U/L	21.5 (17, 31.8)	20 (17, 27)	0.079
ALT, U/L	21.5 (14.3, 34)	19 (14, 27)	0.182
cTnI, ng/mL	0.016 (0.012, 0.180)	0.012 (0.012, 0.025)	0.002
TC, mmol/L	4.75 (3.95, 5.70)	4.78 (4.07, 5.55)	0.827
TG, mmol/L	1.45 (1.04, 1.95)	1.38 (1.06, 2.06)	1.000
HDL-C, mmol/L	1.05 (0.89, 1.16)	1.07 (0.93, 1.28)	0.201
LDL-C, mmol/L	2.61 (2.05, 3.30)	2.61 (2.04, 3.35)	0.868
Lipoprotein(a), mg/dL	33.3 (13.2, 51.4)	18.25 (7.7, 44.9)	0.006
LVEF, %	59 (48.3, 60)	60 (57, 61)	0.206
Gensini score	63.5 (35.3, 89)	23.5 (5.3, 50)	<0.001
ACS classification, *n* (%)			<0.001
X syndrome	0 (0)	31 (13.8)	
STEMI	9 (11.8)	26 (11.6)	
NSTEMI	22 (29.0)	24 (10.7)	
UA	45 (59.2)	143 (63.8)	

Continuous variables with non-normal distribution are presented as median (P25, P75); categorical variables are presented as *n* (%). Abbreviations: BMI, body mass index; SBP, systolic blood pressure; DBP, diastolic blood pressure; WBC, white blood cell; AST, aspartate aminotransferase; ALT, alanine aminotransferase; cTnI, cardiac troponin I; TC, total cholesterol; TG, triglyceride; HDL-C, high-density lipoprotein cholesterol; LDL-C, low-density lipoprotein cholesterol; LVEF, left ventricular ejection fraction; ACS, acute coronary syndrome; STEMI, ST-segment elevation myocardial infarction; NSTEMI, non-ST-segment elevation myocardial infarction; UA, unstable angina.

**Table 2:** Univariate and multivariate Cox regression analysis for adverse events.

Variable	Univariate Analysis		Multivariate Analysis *	
	HR (95% CI)	*p* Value	HR (95% CI)	*p* Value
Age (per 1 year)	1.008 (0.986–1.030)	0.497	1.019 (0.992–1.048)	0.168
Male (vs. female)	2.115 (1.233–3.628)	0.007	1.683 (0.850–3.334)	0.136
Smoking (yes vs. no)	1.760 (1.096–2.828)	0.019	1.665 (0.899–3.082)	0.105
Hypertension (yes vs. no)	0.886 (0.565–1.389)	0.598	0.721 (0.425–1.223)	0.225
Diabetes (yes vs. no)	1.407 (0.837–2.365)	0.198	1.335 (0.672–2.652)	0.410
WBC (per 1 × 10^9^/L)	1.112 (1.016–1.216)	0.022	–	–
Creatine kinase (per 1 U/L)	1.001 (1.000–1.001)	0.008	–	–
Lipoprotein(a) (per 1 mg/dL)	1.013 (1.004–1.022)	0.004	1.021 (1.011–1.031)	<0.001
LVEF (per 1%)	0.962 (0.932–0.993)	0.018	–	–
Gensini score (per 1 unit)	1.014 (1.009–1.018)	<0.001	1.016 (1.010–1.022)	<0.001

* Multivariate model adjusted for age, sex, smoking, hypertension, diabetes, SBP, DBP, hospital stay, WBC, hemoglobin, platelet, D-dimer, uric acid, albumin, AST, ALT, GGT, cTnI, CK, LDH, GG, TG, apo A1, LVEF, Gensini score, with exclusion of collinear variables (VIF > 5). Abbreviations: WBC, white blood cell; LVEF, left ventricular ejection fraction; ACS, acute coronary syndrome; HR, hazard ratio; CI, confidence interval.

**Table 3:** Subgroup analysis for the association between lipoprotein(a) and adverse events.

Subgroup	HR * (95% CI)	*p* Value	*p* for Interaction
Sex			0.669
Female	1.023 (0.998–1.049)	0.076	
Male	1.023 (1.007–1.039)	0.004	
Age			0.889
<60 years	1.024 (1.004–1.045)	0.021	
≥60 years	1.021 (1.005–1.038)	0.013	
ACS subtype			0.621
STEMI	0.961 (0.882–1.047)	0.365	
NSTEMI	1.539 (0.894–2.649)	0.119	
UA	1.023 (1.006–1.040)	0.007	

* HR represents the risk per 1 mg/dL increase in lipoprotein(a), adjusted for traditional risk factors and Gensini score. Abbreviations: ACS, acute coronary syndrome; STEMI, ST-segment elevation myocardial infarction; NSTEMI, non-ST-segment elevation myocardial infarction; UA, unstable angina; HR, hazard ratio; CI, confidence interval.

**Table 4:** IDI analysis for incremental value of Lp(a).

Model Comparison	IDI (95% CI)	*p* Value
Base model vs. Base + Lp(a)	0.0172 (−0.0068–0.0400)	0.180

Footnote: The base model included age, sex, hypertension, diabetes, smoking, lipid profiles, and Gensini score. Abbreviations: IDI, integrated discrimination improvement; CI, confidence interval.

## Data Availability

The datasets generated during and/or analyzed during the current study are not publicly available due to privacy or ethical restrictions, but are available from the corresponding author on reasonable request.

## Abbreviations

The following abbreviations are used in this manuscript:

ACS	Acute coronary syndrome
PCI	Percutaneous coronary intervention
CABG	Coronary artery bypass grafting
MACE	Major adverse cardiovascular events
Lp(a)	Lipoprotein(a)
ASCVD	Atherosclerotic cardiovascular disease
CAG	Coronary angiography
MI	Myocardial infarction
ROC	Receiver operating characteristic
DCA	Decision curve analysis
IDI	Integrated discrimination improvement
CHD	Coronary atherosclerotic heart disease
LDL	Low-density lipoprotein
ApoB-100	Apolipoprotein B-100
Apo A1	Apolipoprotein A1
BMI	body mass index
SBP	systolic blood pressure
DBP	diastolic blood pressure
WBC	white blood cell
ALT	Alanine aminotransferase
AST	Aspartate aminotransferase
cTnI	cardiac troponin
TC	Total cholesterol
TG	Triglycerides
HDL-C	High-density lipoprotein cholesterol
LDL-C	Low-density lipoprotein cholesterol
TRIPOD	Transparent Reporting of a multivariable prediction model for individual prognosis or diagnosis
LVEF	Left ventricular ejection fraction
SD	Standard deviation
VIF	Variance inflation factor
AUC	Area under the curve
CI	Confidence interval
HR	Hazard ratio
UA	Unstable angina
NSTEMI	Non-ST-Elevation Myocardial Infarction
STEMI	ST-Elevation Myocardial Infarction
MENZACS	Multi-Ethnic Study of Acute Coronary Syndromes
MESA	Multi-Ethnic Study of Atherosclerosis
PCSK9i	Proprotein convertase subtilisin/kexin type 9 inhibitors
